# Detection of melanoma, breast cancer and head and neck squamous cell cancer sentinel lymph nodes by Tc-99m Tilmanocept (Lymphoseek®)

**DOI:** 10.1007/s10585-021-10137-4

**Published:** 2021-12-28

**Authors:** Stanley P. Leong

**Affiliations:** 1 California Pacific Medical Center and Research Institute, San Francisco, CA USA; 2 University of California School of Medicine San Francisco, San Francisco, CA USA

**Keywords:** Tc-99m-labeled Tilmanocept, Lymphoseek®, Sentinel lymph nodes, Melanoma, Breast cancer, Head and neck squamous cell carcinoma

## Abstract

Technetium-99m-labeled Tilmanocept or Lymphoseek® (Cardinal Health, Dublin, Ohio) is a soluble, synthetic molecule with a small diameter (7 nm), which is comprised of technetium-99m chelated to a dextran backbone containing multiple units of mannose ligands with a high affinity for CD206, a receptor located on the surface of macrophages and dendritic cells that are found in high concentration in lymph nodes. It enables quick transit from the injection site and rapid lymph node accumulation. The binding of mannose ligand and CD206 results in the internalization of the ligand and receptor into the cell. Once the Technetium-99m-labeled Tilmanocept (Lymphoseek®) reaches the lymph node, it is readily internalized by the macrophages and dendritic cells within the draining lymph nodes. Technetium-99m-labeled Tilmanocept (Lymphoseek®) has been extensively studied as a radioisotope for detection of sentinel lymph nodes in melanoma, breast cancer and head and neck squamous cell carcinoma in clinical trials. Based on its safety and ability to detect sentinel lymph nodes satisfactorily, it has been approved by the FDA to use as a radioisotope for preoperative lymphoscintigraphy for identification of sentinel lymph nodes in these types of cancer. Further, the FDA has expanded approval of Technetium-99m-labeled for sentinel lymph node mapping of all solid tumors as well as in pediatric patients.

## Introduction

The concept of sentinel lymph node biopsy (SLNB) to stage the regional lymph node with avoidance of a traditional radical lymph node dissection has been developed by the work of two SLN surgical pioneers, Cabanas [1] with the penile carcinoma model based on the anatomical location of SLNs and Morton [2] with the melanoma model based on the lymphatic drainage of the primary site to the SLNs. Subsequent clinical trials in melanoma [3] and breast cancer [4] have firmly established that SLNB is a reliable staging procedure for the regional nodal basin. The significance of the SLN concept is that a radical lymph node dissection with increased morbidity can be spared if the SLNB is negative. Although blue dye or isosulfan blue (Lymphazurin™) was used in the original study for melanoma SLN procedure [2], the critical development was the adoption of radiotracer [5, 6] to identify SLNs. Technetium-99m sulfur colloid was widely used as it serves two major goals: (1) the ability to define the drainage patterns of the primary cancer site such as melanoma [6] or breast cancer to the regional nodal basin preoperatively and (2) facilitation of intraoperative identification of SLNs using a gamma probe [5].

**Fig. 1:**
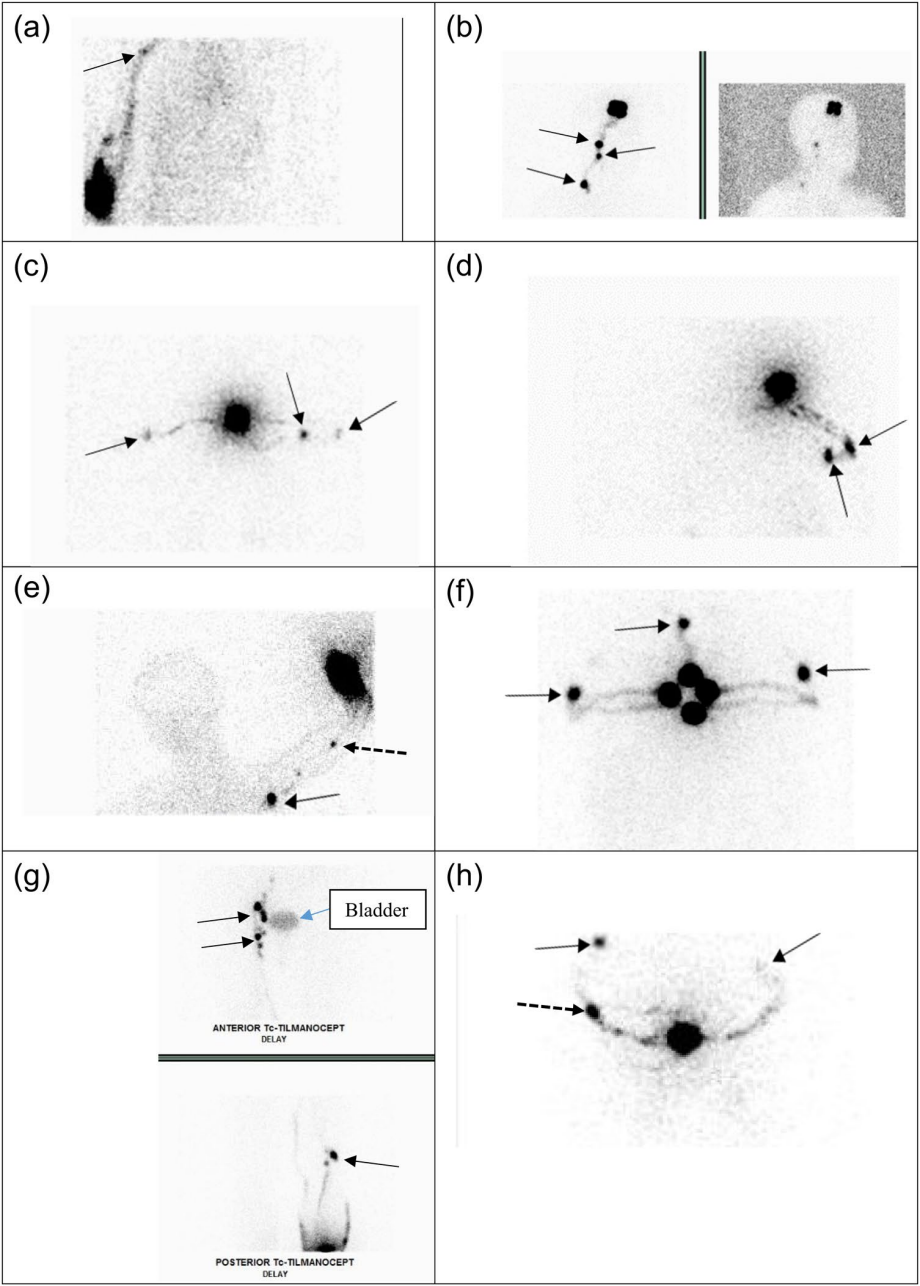
Pre-operative lymphoscintigraphy demonstrates varying lymphatic channel patterns in patients with primary melanoma using Technetium-99m-labeled Tilmanocept (Lymphoseek®). Straight arrow = sentinel lymph node; dashed arrow = in-transit sentinel lymph node. **A** Drainage of a single channel from the right upper arm leading to one SLN in the right axilla. **B** Drainage of a single channel from a right parietal scalp lesion to multiple contiguous nodes in the right neck. **C** Confluent right channels drain from the upper back to two SLNs in the right axilla and a single channel draining to a single SLN in the left axilla. **D** Multiple channels from the right upper back draining to two SLNs in the right axilla. **E** Multiple channels from the left proximal forearm, leading to a single epitrochlear SLN and a single SLN in the left axilla. **F** Multiple drainage channels from a lesion in the anterior chest wall to SLNs in both axillae and one single SLN in the supraclavicular node at the suprasternal notch. **G** Multiple channels from the right lower extremity draining to multiple SLNs in the pelvic, femoral, and popliteal basin. Bladder activity is present in the image. **H** Multiple channels from the midline back draining to multiple SLNs in different basins, one in the right and one in the left axilla and a single lateral left upper back in-transit SLN

Preoperative lymphoscintigraphy is mandatory for determining the SLN basin(s) for primary melanoma as it may arise from any site of the body. Singular or multiple nodal basins may be detected. For example, a primary melanoma in the distal lower extremity, it may drain to the popliteal basin, in addition to the inguinal basin. A lesion in the upper extremity, it may drain to the epitrochlear basin, in addition to the axillary basin. A truncal primary melanoma may drain to multiple sites including the lower neck, bilateral axillae and groins. On the other hand, a primary breast cancer usually drains to the ipsilateral axilla. It is mandatory to have preoperative lymphoscintigraphy to map out the exact locations of the nodal basins draining the primary melanoma site. Figure 1 shows the various patterns of lymphatic drainage from melanoma of different anatomic sites to different nodal basin.

In the 1990s, when SLNB was being developed, both Lymphazurin™ and radiotracer were used as the blue dye added the visualization of the SLN being blue. The identification of sentinel nodes was reported to be increased to 97% to 99% [7, 8]. To date, radiotracer such as Tc-99m sulfur colloid is used to identify the SLN basins by lymphoscintigraphy and to detect the SLNs by a gamma probe intraoperatively and when used alone, successful mapping of SLNs may result in localization over 95–98% [9, 10].

In this review article, the identification of SLNs in melanoma, breast cancer and head and neck cancer using Technetium-99m-labeled Tilmanocept (Lymphoseek®) will be discussed in detail. The content of this review is based on the presentations by Stanley Leong on the identification of melanoma and breast cancer SLNs and by Stephen Lai on the identification of head and neck cancer SLNs using Lymphoseek during a mini-symposium being sponsored by Cardinal Health (Dublin, OH).

## Characteristics of Technetium-99m-labeled Tilmanocept

**Fig. 2:**
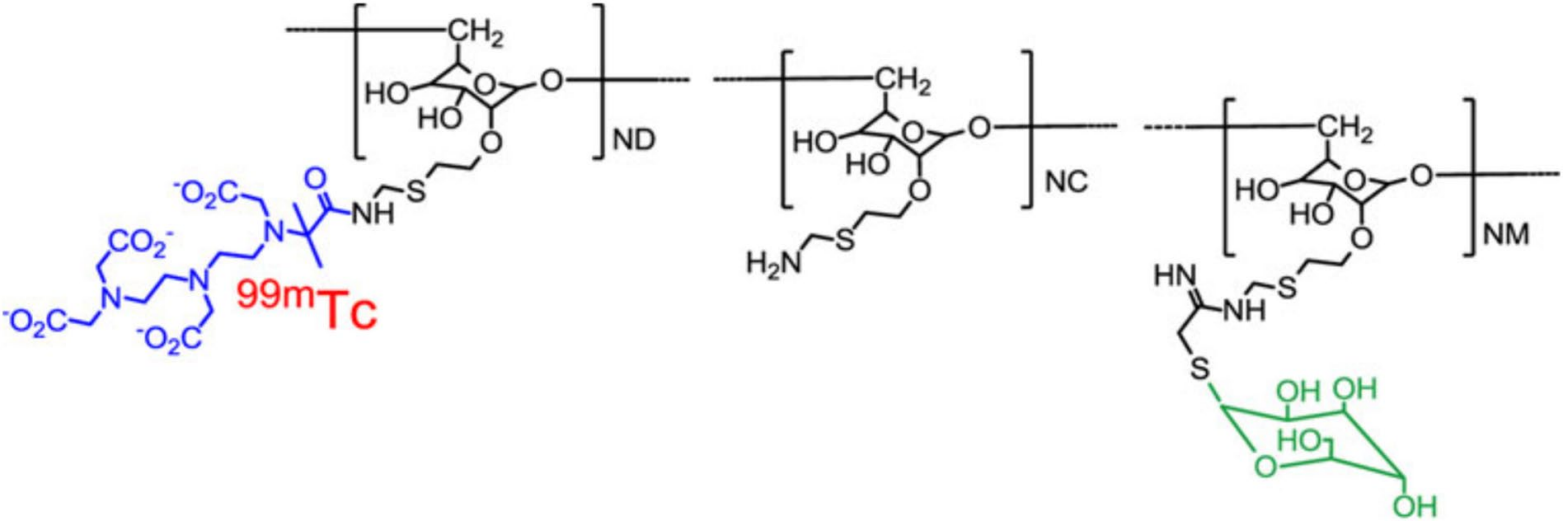
The chemical structure of Tc-99m tilmanocept consists of a dextran backbone (black) to which multiple units of mannose (green) and DTPA (blue) are attached. The mannose units provide a molecular mechanism by which Tc-99m tilmanocept binds are attached avidly to a receptor specific to reticuloendothelial cells (CD206). The DTPA units provide a highly stable means to radiolabel tilmanocept with ^99m^Tc (red). The molecular weight of Tc-99m tilmanocept is approximately 19,000 g/mol; the molecular diameter is 7.1 nm. Figure and legend reprinted by permission from Springer: Annals of Surgical Oncology, Comparative evaluation of Tc-99m Tilmanocept for sentinel lymph node mapping in breast cancer patients: Results of two phase 3 trials, Wallace et al. 2013 [18]. (Color figure online)

**Fig. 3:**
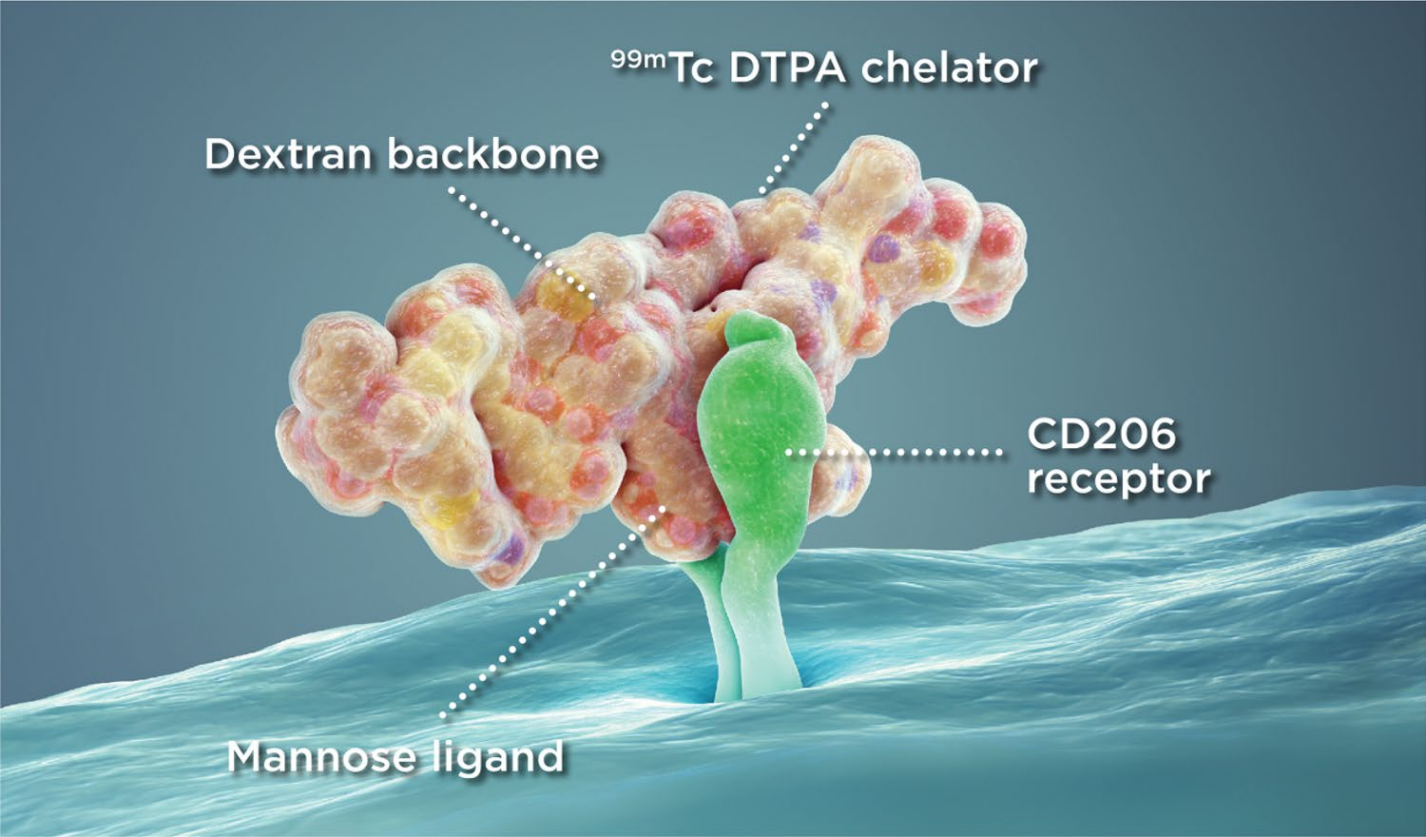
Illustration of the binding of mannose ligand of Lymphoseek with the CD206 receptor on the macrophages or dendritic cells in the lymph nodes. Reprinted from www.lymphoseek.com. (Color figure online)

We and others have studied a novel radiotracer, Technetium-99m-labeled Tilmanocept (Lymphoseek®), to identify SLNs [11, 12]. Technetium-99m-labeled Tilmanocept (Lymphoseek®) is a soluble, synthetic molecule with a small diameter (7 nm) that enables rapid transit from the injection site and rapid lymph node accumulation. Technetium-99m-labeled Tilmanocept (Lymphoseek®) is chelated to the dextran backbone, which also has mannose ligands (Fig. 2) with a high affinity (equilibrium binding constant of 0.12 nmol/L) for and bind tightly to CD206, a receptor located on the surface of macrophages and dendritic cells that is found in high concentration in lymph nodes [13–15]. The binding of mannose ligand and CD206 results in the internalization of the ligand and receptor into the cell. Once the Technetium-99m-labeled Tilmanocept (Lymphoseek) reaches the lymph node, it is readily internalized by the macrophages and dendritic cells within the draining lymph nodes (Fig. 3).

The traditional usage of Tc-99m Sulfur Colloid is compared with Technetium-99m-labeled Tilmanocept (Lymphoseek®) in Table 1. The major differences between the two radiotracers are: (1) Technetium-99m-labeled Tilmanocept (Lymphoseek®) binds with the receptor of the macrophages and dendritic cells and (2) it has a homogeneous size.

**Table 1:** Characteristics of Tc-99m Sulfur Colloid and Tc-99m-labeled Tilmanocept (Lymphoseek®)

	Filtered Tc-99m Sulfur Colloid	Unfiltered Tc-99m Sulfur Colloid	Tc-99m Tilmanocept
Availability	Widely available with all commercial U.S. radiopharmacies	Widely available with all commercial U.S. radiopharmacies	Cardinal Health has exclusive rights in the US. Widely available across 130 + Cardinal radiopharmacies
Cost	Charged for each single injection. Comparable with tilmanocept for multiple unit dose administrations	Charged for each single injection. Comparable with tilmanocept for multiple unit dose administrations	Charged as cost per patient, independent of # of syringes or activity ordered
Particle size	24 nm [35]	Two sizes: 2.5 & 54 nm [35]	Not a particle. Molecular size is 7 nm and homogeneous
Product stability (by package insert)	Grows in size at 6 h Not FDA-approved (no package insert) [36]	6 h	6 h
Injection Site Clearance (half-life)	24 h [37]	57 h [38]	2.6 h [37]
Lymph node accumulation	Phagocytosis of macrophages or retention due to particle size. Passive accumulation, and will travel to additional nodes besides the sentinel node	Phagocytosis of macrophages or retention due to particle size. Passive accumulation, better sentinel node retention than filtered Tc-SC	Receptor binding to mannose (CD206) receptors
Extra-SLN clearance	Phagocytized by Kupffer cells in liver, spleen and bone marrow	Phagocytized by Kupffer cells in liver, spleen and bone marrow	Renal mesangial cells; endothelial lining cell, and Kupffer cells in the liver
Body background	None, accumulation mainly in liver	None, accumulation mainly in liver	Minimal soft tissue activity, mild liver, renal and bladder activity from clearance
Pain and discomfort	Can be painful due to acidic nature of sulfur colloid (pH 5.0–5.5). Buffered Lidocaine can reduce acidity to pH 6.4–6.8	Can be painful due to acidic nature of sulfur colloid (pH 5.0–5.5). Buffered Lidocaine can reduce acidity to pH 6.4–6.8	Non-Acidic (pH 6.8–7.2). Lidocaine and buffering not required
Radiation dosimetry (total body)	Less than 0.016 rad/mCi	0.016 rad/mCi	0.011 rad/mCi

The cost of radiopharmaceuticals can vary from institution to institution, as the actual cost is based on the individual contracts issued between the distributor (radiopharmacy) and the end user. Lymphoseek® is a product specifically designed for lymphatic mapping and carries a higher cost per dose than the off-label application of technetium sulfur colloid. Lymphoseek® is ready to be used upon delivery by Cardinal Health and is charged as a per patient dose ($500), regardless of how many syringes are dispensed. Sulfur colloid can be charged as a per unit dose ($100) or per patient dose depending on the individual contract and user preference. While our institution (California Pacific Medical Center) prefers four individual unit does injections of Lymphoseek® ($500 per patient), sulfur colloid wound cost $400. Other institutions may prefer to administer the radiopharmaceutical one injection using only one syringe ($100). This technique could significantly reduce the cost of sulfur colloid by a factor of four. Other cost containment practices include the in-house preparation and filtration of technetium sulfur colloid for multi-dose, multiple patient dispensing. In this time of <USP825> compliance, additional costs of clean hood, clean area and staff competency needs to be considered

## Use of Technetium-99m-labeled Tilmanocept (Lymphoseek) in specific populations

There are no data available on Technetium-99m-labeled Tilmanocept (Lymphoseek®) use in pregnant women. However, a fetal outcome after technetium scintigraphy in early pregnancy showed no teratogenic effects as compared to a group of pregnant women without exposure [16]. Therefore, it can be assumed that either Tc-99m Sulfur Colloid or Technetium-99m-labeled Tilmanocept (Lymphoseek®) is safe for pregnant women especially post first trimester.

If considering Technetium-99m-labeled Tilmanocept (Lymphoseek®) administration to be used in the identification of SLNs in a pregnant woman, the patient should be informed about the potential for adverse pregnancy outcomes based on the radiation dose from the drug and the gestational period of the pregnancy. Regarding Technetium-99m-labeled Tilmanocept (Lymphoseek® administration to a lactating woman, it is advised to pump and discard breast milk for 24–48 h after injection to decrease radiation exposure to the breastfed child. Prescribing recommendations for Lymphoseek® may be found in the website, https://www.accessdata.fda.gov/drugsatfda_docs/label/2016/202207s005lbl.pdf.

## Lymphoscintigraphy imaging following Technetium-99m-labeled Tilmanocept (Lymphoseek) injection

**Fig. 4:**
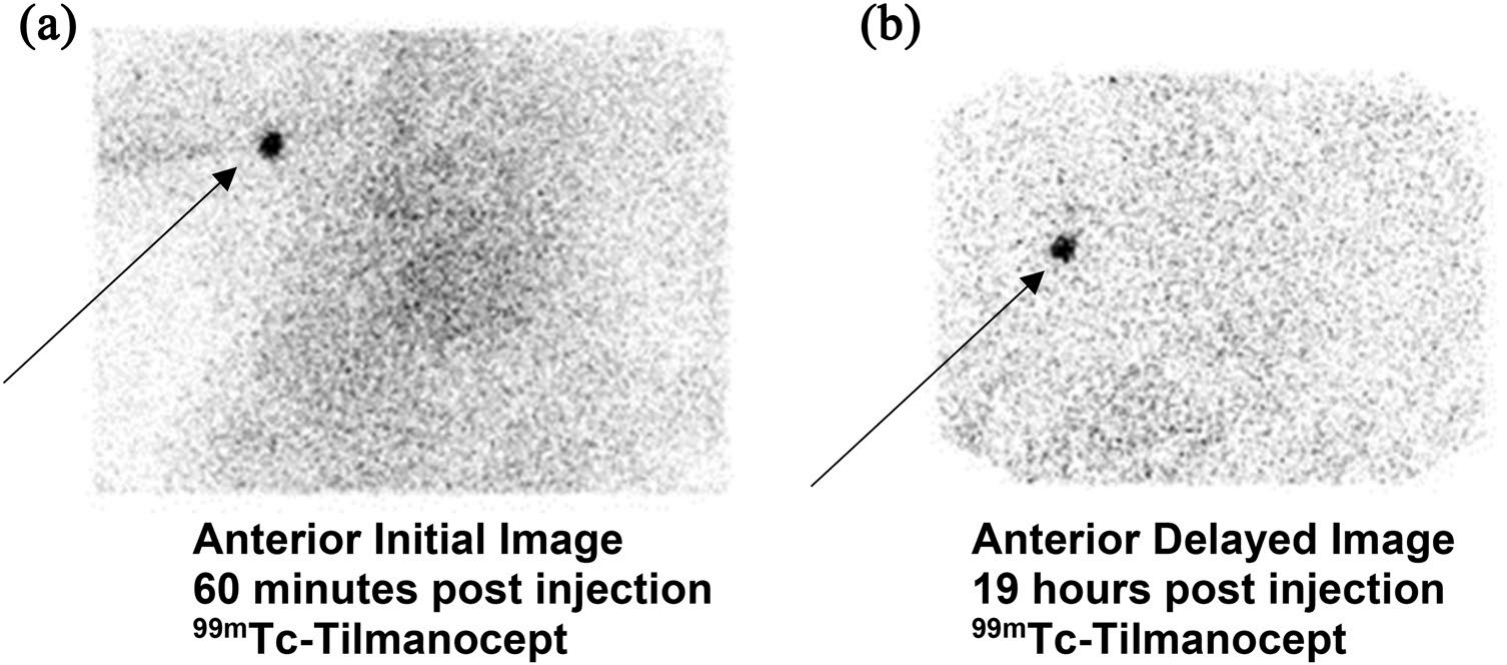
**A** and **B** 76-year-old Caucasian gentleman with *melanoma at least 1.2 mm*, nonulcerated, with 2 mitoses/mm^2^ extending focally to the specimen base on his right forearm. He underwent lymphoscintigraphy consisting of four injections of 0.1 mL, 500 uCi of Tc99m-Tilmanocept aliquots around the biopsy site. Imaging was performed immediately after injection, consisting of dynamic planar acquisitions. Additional planar imaging was performed on the day of surgery, 19 h after injection showing no change in the localization of the right axillary sentinel lymph node

Technetium-99m-labeled Tilmanocept (Lymphoseek®) has been shown to have a rapid lymphatic uptake to first-echelon lymph nodes with high accumulation within the nodes. Although it is assumed that there is minimal pass-through to second-echelon nodes (www.Lymphoseek.com), the definitive study to compare immediate post-injection lymphoscintigraphy and delayed (18–24 h post-injection) lymphoscintigraphy in a series of patients has not been done. Nevertheless, the following case shows no difference between the immediate and 24 h lymphoscintigraphy images (Fig. 4) showing no migration of Tc-99m Tilmanocept (Lymphoseek®) within 24 h following injection.

## Phase 2 Technetium-99m-labeled Tilmanocept melanoma and breast cancer sentinel lymph node clinical study

In a prospectively planned and open-label phase 2 clinical study in five US sites, the primary objective of the study was to determine the preoperative lymphoscintigraphic identification of SLNs draining the primary site of melanoma or breast cancer with Technetium-99m-labeled Tilmanocept (Lymphoseek®) used as a radiotracer and intraoperative gamma probe localization of SLNs in the regional nodal basin. Institutional review board approval was obtained in each site and the U.S. IND regulations (21CFR 56) were strictly followed.

Each patient received 50 μg (2.6 nmol) of Technetium-99m-labeled Tilmanocept (Lymphoseek®), radiolabeled with either 0.5 mCi for same-day surgery or 1.0 mCi for next-day surgery, injected near the primary tumor, followed by intraoperative lymphatic mapping. The interval between injection and SLN surgery ranged from 15 min to 24 h, depending on the surgical schedule. Lymphazurin™ injection at surgery was optional as a visual aid.

A handheld gamma probe was used to detect the SLN intraoperatively. Forty-seven patients with melanoma 31 patients with breast cancer (78 total patients) were evaluable in the study. For those whom lymphoscintigraphy was performed (55 patients, mostly with melanoma, 70.5%), a Technetium-99m-labeled Tilmanocept (Lymphoseek®) hot spot was identified in 94.5% of patients before surgery. During surgery, Technetium-99m-labeled Tilmanocept (Lymphoseek®) identified at least one regional SLN in 75 (96.2%) of 78 patients: 46 of 47 (97.9%) in melanoma and 29 of 31 (93.5%) in breast cancer cases. Tissue specificity of Technetium-99m-labeled Tilmanocept (Lymphoseek®) for lymph nodes was 100% with 95.1% mapping sensitivity by localizing in 173 of 182 nodes removed from surgery. The overall rate of metastatic disease in the Technetium-99m-labeled Tilmanocept (Lymphoseek®)-identified nodes was 13.7%. Five procedure-related serious adverse events occurred; none was related to Technetium-99m-labeled Tilmanocept (Lymphoseek®). This phase 2 study has demonstrated the safety and efficacy of Technetium-99m-labeled Tilmanocept (Lymphoseek®) for use in SLN mapping. Based on the high intraoperative localization of SLNs and lymph node specificity of Technetium-99m-labeled Tilmanocept (Lymphoseek®), as well as the identification of metastatic disease within the nodes, it can be concluded that SLNs from melanoma and breast cancer may be accurately identified by this novel mannose receptor-targeted molecule [12].

## Phase 3 Technetium-99m-labeled Tilmanocept (Lymphoseek®) melanoma sentinel lymph node clinical study

**Fig. 5:**
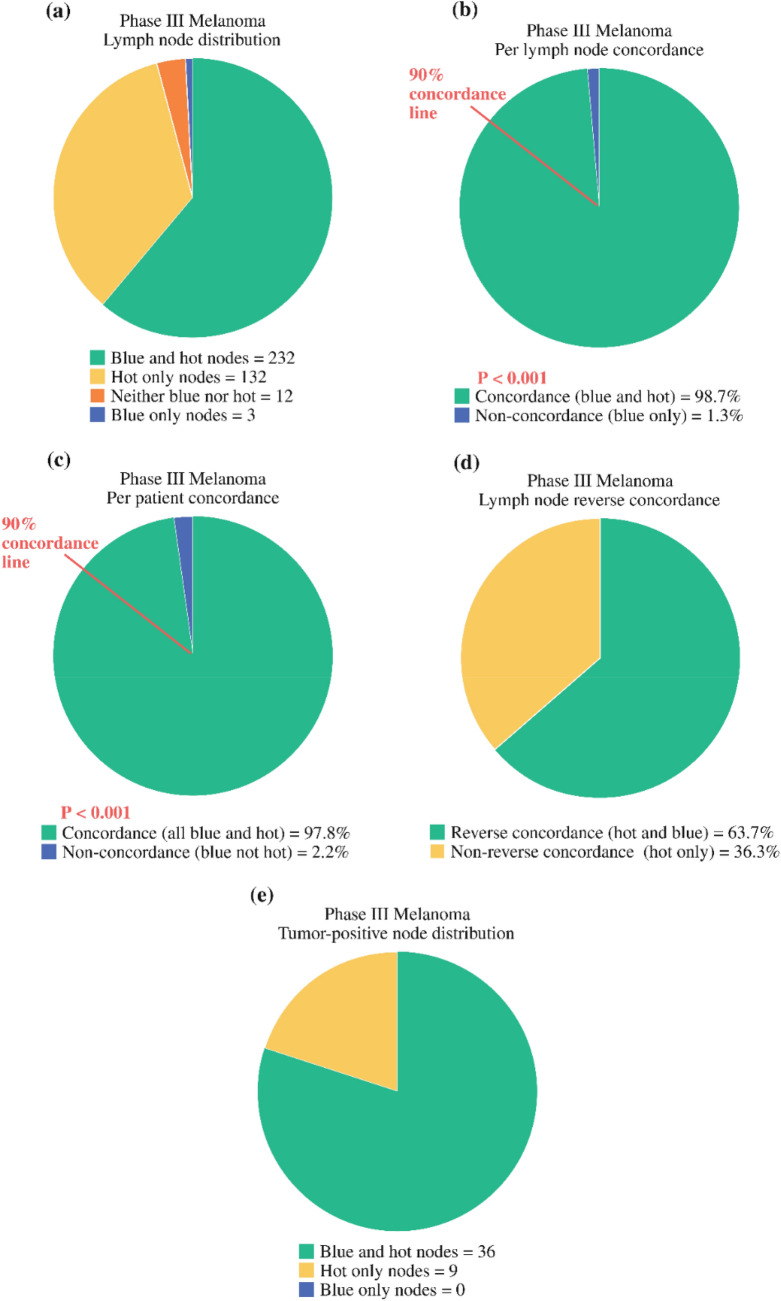
Distribution of resected lymph nodes and concordance of Tc-99m tilmanocept with blue dye. **a** Analysis of 379 excised lymph nodes; 12 lymph nodes removed from 9 patients were not blue and their radioactivity did not meet the protocol criteria above background; all were negative for metastatic melanoma. **b** Lymph node concordance: on the basis of a total of 235 blue lymph nodes. The statistical level for concordance was prospectively determined that 90% of blue nodes would be hot. (In **b** and **c**, the red line marks the 90% concordance cutoff level. **c** Patient concordance: based on 138 patients with at least one lymph node to be blue. **d** Lymph node reverse concordance: on the basis of a total of 364 hot nodes. Only 232 were also blue (63.7%). **e** Distribution of lymph node containing micrometastasis: on the basis of 45 lymph nodes containing melanoma by routine histology and/or immunohistochemistry. All blue lymph nodes with melanoma were also hot. Figure and legend reprinted with permission from Springer: Annals of Surgical Oncology, Combined analysis of phase III trials evaluating ^99m^Tc Tilmanocept and Vital Blue Dye for identification of sentinel lymph nodes in clinically node-negative cutaneous melanoma, Sondak et al. 2013. [17]. (Color figure online)

Following the Phase 2 study, a multicenter Phase 3 study was carried out to compare the identification of SLNs in melanoma patients between Technetium-99m-labeled Tilmanocept (Lymphoseek®) and blue dye. Technetium-99m-labeled Tilmanocept (Lymphoseek®) and blue dye were injected in the melanoma patients being enrolled in this study. SLNs being identified either by radioactive and/or blue dye intraoperatively were resected and submitted for histologic examination. Concordance as defined by the proportion of blue nodes identified by Technetium-99m-labeled Tilmanocept (Lymphoseek®) was the end point. The prespecified minimum concordance level was 90%. Reverse concordance being defined by the proportion of radioactive nodes detected by blue dye was also calculated. The prospective statistical calculations combined the concordance data from both tracers. Fifteen centers primarily in the United States enrolled 154 melanoma patients who were injected with both agents and were intraoperatively identified by a hand-held gamma probe and visualization of blue coloration in the SLNs. Intraoperatively, 232 of 235 blue nodes were detected by Technetium-99m-labeled Tilmanocept (Lymphoseek®) with a concordance rate of 98.7% (p < 0.001). Of the 364 nodes identified by Technetium-99m-labeled Tilmanocept (Lymphoseek®), the reverse concordance rate was 63.7% (232 of 364 nodes). At least one node in more patients (n = 150) was detected by Technetium-99m-labeled Tilmanocept (Lymphoseek®) than blue dye (n = 138, p = 0.002). In 135 of 138 patients with at least one blue node, all blue nodes also were identified by Technetium-99m-labeled Tilmanocept (Lymphoseek®) with significant radioactivity. Metastatic melanoma was identified in the SLNs of 22.1% of patient. All 45 melanoma-positive SLNs were detected by Technetium-99m-labeled Tilmanocept (Lymphoseek®), however, blue dye identified only 36 of 45 nodes (80%; p = 0.004). No positive SLNs were detected only by blue dye. Four of 34 node-positive patients were identified exclusively by Technetium-99m-labeled Tilmanocept (Lymphoseek®), so 4 of 154 patients (2.6%) were correctly staged only by Technetium-99m-labeled Tilmanocept (Lymphoseek®). No serious adverse events resulted from Technetium-99m-labeled Tilmanocept Lymphoseek®) injection. Thus, it was concluded that Technetium-99m-labeled Tilmanocept (Lymphoseek®) met the prespecified primary concordance end point, identifying 98.7% of blue nodes. More importantly, it identified more SLNs in more patients, and detected more melanoma-containing nodes than blue dye as shown in Fig. 5 [17].

## Phase 3 Technetium-99m-labeled Tilmanocept (Lymphoseek®) breast cancer sentinel lymph node clinical study

**Fig. 6:**
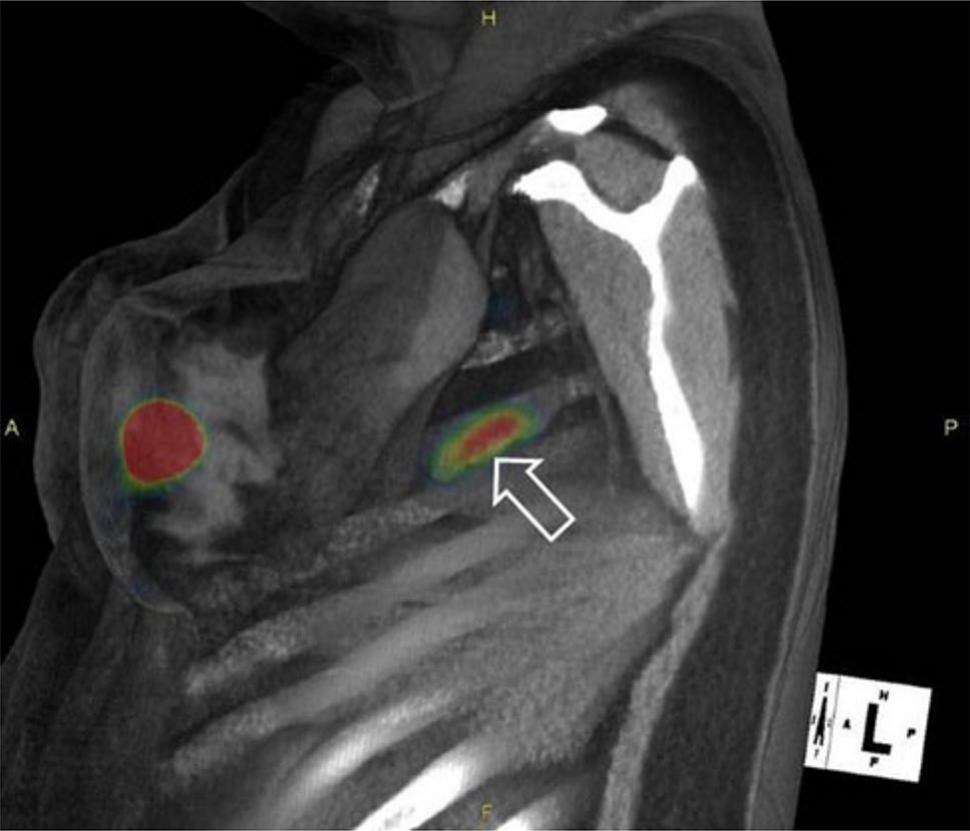
Lymphoscintigraphy of a 35-year-old woman with carcinoma in situ of the left breast showing 2 intense foci of noted 99mTc tilmanocept localization within the left axilla. An intradermal injection (0.4 mL, 0.5 mCi, 3.0 nmol) of 99mTc tilmanocept was administered to the upper left quadrant of the left breast. The SPECT/CT image is a fused sagittal cross section acquired 1 h postinjection, which visualizes a sentinel lymph node (arrow) and the injection site. At 5 h after injection, 3 blue and hot lymph nodes (6724 cps, 1477 cps, 167 cps) were detected at surgery and excised. Pathologic examination revealed 1 histologically positive lymph node (blue with 6700 cps, 1.7 9 1.3 9 0.7 cm) and 2 negative lymph nodes. Figure and legend reprinted by permission from Springer: Annals of Surgical Oncology, Comparative evaluation of 99mtc tilmanocept for sentinel lymph node mapping in breast cancer patients: Results of two phase 3 trials, Wallace et al. 2013 [18]. (Color figure online)

In a separate phase 3 study for breast cancer, the same protocol was adopted for patients with primary breast cancer [18]. Thirteen centers enrolled 148 breast cancer patients. Again, each patient received Technetium-99m-labeled Tilmanocept (Lymphoseek®) and blue dye. The primary endpoint of concordance was the same as the lower boundary set point of 90% with respect to the proportion of lymph nodes detected by blue dye and Technetium-99m-labeled Tilmanocept (Lymphoseek®). Intraoperatively, 207 of 209 nodes identified by blue dye were also detected by Technetium-99m-labeled Tilmanocept (Lymphoseek®) with a concordance rate of 99.04% (p < 0.0001). A total of 320 lymph nodes were detected by Technetium-99m-labeled Tilmanocept (Lymphoseek®) of which 207 (64.7%) were detected by Lymphazurin. Technetium-99m-labeled Tilmanocept (Lymphoseek®) detected at least 1 SLN in more patients (146) than did Lymphazurin (131, p < 0.0001). In 129 of 131 patients with ≥ 1 blue node, all blue nodes were also radioactive. Of 33 lymph nodes with metastatic breast cancer (18.2% patient positivity rate), Technetium-99m-labeled Tilmanocept (Lymphoseek®) detected 31 of 33, whereas blue dye identified only 25 of 33 (p = 0.0312). No positive SLNs were detected only by blue dye. Again, no serious adverse events were noted from Technetium-99m-labeled Tilmanocept (Lymphoseek®). Thus, the study concluded that Technetium-99m-labeled Tilmanocept (Lymphoseek®) had met the primary endpoint with concordance rate of 99.04%. Further, Technetium-99m-labeled Tilmanocept (Lymphoseek®) was able to identify more SLNs in more patients and more lymph nodes with metastatic breast cancer than blue dye [18]. Figure 6 shows lymphoscintigraphy with Technetium-99m-labeled Tilmanocept (Lymphoseek®) of a 35-year-old woman with carcinoma in situ of the left breast showing 2 intense foci of radiotracer localization within the left axilla.

Overall from the clinical trial experience on patients over age 18, less than 1% of patients noted injection site irritation and/or pain after Technetium-99m-labeled Tilmanocept (Lymphoseek) administration [1]. No patients experienced serious adverse reactions following Technetium-99m-labeled Tilmanocept (Lymphoseek) injection. The safety and effectiveness of Technetium-99m-labeled Tilmanocept (Lymphoseek) additionally have been established in pediatric patients 1 month of age and older (www.lymphoseek.com).

## Sentinel lymph nodes in head and neck squamous cell carcinoma (HNSCC)

Like melanoma and breast cancer, lymph node metastasis is one of the most important prognostic factors in HNSCC [19]. Over the past 4 decades, for patients with T1–2, N0 clinical staging, there has been an evolution of observation, elective neck dissection and SLNB [20, 21]. Similar to melanoma and breast cancer, the advantages of a SLN biopsy in HNSCC are: (1) directing pathologic analysis on nodes most likely to harbor micrometastasis, (2) potentially decreasing surgical morbidity and (3) localization of unexpected patterns of lymphatic drainage in the contralateral neck [22–24]. Thus, in this group of patients, with about 20% of occult metastasis in the cervical nodes, SLN biopsy can identify these patients for an elective neck dissection and spare about 80% of patients of a neck dissection with a negative SLN biopsy.

In 2010, the American College of Surgeons Surgical Oncology Group (ACOSOG) trial Z0360 was published on 106 patients with T1–2 oral cavity squamous cell carcinoma with no clinical adenopathy, recruited from 25 US institutions [25] as a Phase II study with preoperative lymphoscintigraphy using unfiltered Tc-99m sulfur colloid within 18 h of the surgical procedure, SLNB and simultaneous completion lymph node dissection of the neck. In this group of patients, 100 patients were found to have no other pathologically positive nodes using hematoxylin and eosin stain with a negative-predictive value of 94%. Additional sectioning and immunohistochemistry improved the negative-predictive value to 96%. The true-positive rate in the 40 patients with positive cervical lymph nodes was 90.2% being superior for tongue cancer relative to floor of mouth. Metastases were correctly identified in 100% of the T1 lesions. The authors concluded that for T1 or T2 N0 oral squamous cell carcinoma performed by surgeons of various experience levels accurately predicted a pathologically negative neck in 96% of patients by SLNB with step sectioning and immunohistochemistry. In a separate study in Europe, the SENT trial recruiting 415 patients with T1–2 N0 squamous cell carcinoma of the mouth with a follow-up of 3 years, using nanocolloid for lymphoscintigraphy, has found a positive SLNB rate of 23% with a negative-predictive value of 95%. The false negative rate was 14% with 8 patients being rescued by salvage lymph node dissection. The sentinel node status was significantly correlated with overall survival (p = 0.00013) [26]. In a meta-analysis for diagnostic efficacy of SLNB for early HNSCC over 66 studies with 3566 patients with cT1-2N0 oral squamous cell carcinoma being included in this meta-analysis, the pooled SLN identification rate was 96.3%(95% CI 95.3–97.0%). The pooled sensitivity was 0.87 (95% CI 0.85–0.89), pooled negative predictive value was 0.94 (95% CI 0.93–0.95), and AUC was 0.98 (95% CI 0.97–0.99). In addition, subgroup analyses showed that SLN assessment with immunohistochemistry achieved a significantly higher sensitivity than without immunohistochemistry. The authors conclude that this meta-analysis suggests that SLNB has a high diagnostic accuracy in cT1–2 N0 oral squamous cell carcinoma, and is an ideal alternative to elective neck dissection. Furthermore, the use of IHC can significantly improve SLNB diagnostic sensitivity for early OSCC [27].

Based on these non-randomized studies, the SLN procedure may be a viable alternative to a neck lymph node dissection to assess the cervical nodal basin for patients with T1–2 N0 oral cavity squamous cell carcinoma.

## Technetium-99m-labeled Tilmanocept (Lymphoseek®) for identification of sentinel lymph nodes for head and neck squamous cell carcinoma

Technetium-99m-labeled Tilmanocept (Lymphoseek®) for SLN mapping in HNSCC patients was evaluated in an open-label, nonrandomized, single-arm phase 3 clinical trial with enrollment of 101 patients with T1–4 N0 and M0 HNSCC. The goal of the study was to determine the false negative rate of SLNB with respect to pathologic nodal status with intraoral or cutaneous HNSCC [28]. Technetium-99m-labeled Tilmanocept (Lymphoseek®) performance metrics are shown in Table 2.

**Table 2:** Tc-99m-labeled Tilmanocept (Lymphoseek®) results in HNSCC study

Diagnostic metrics	Rate in % (95% confidence interval)
False negative rate (out of 39 pathology-positive patients)	2.56 (0.06, 13.49)
Negative predictive value (out of 45 true and false negative patients)	97.78 (88.23, 99.94)
Overall accuracy (out of 83 total patients)	98.80 (93.47, 99.97)

LYMPHOSEEK® had a low false negative rate (FNR) in SCC of the oral cavity [28]

As noted in Table 2, the false negative rate with Technetium-99m-labeled Tilmanocept (Lymphoseek®) (2.6%) is much lower compared to 9.8% with sulfur colloid in the ACOSOG study and 14% with nanocolloid in the SENT trial. Based on the published clinical trials on Technetium-99m-labeled Tilmanocept (Lymphoseek®) on melanoma, breast cancer and HSNCC, Technetium-99m-labeled Tilmanocept (Lymphoseek®) has been approved by the FDA for clinical utility for preoperative lymphoscintigraphy and guiding intraoperative SLN biopsy to identify SLNs [29]. In addition, the FDA has expanded approval of Technetium-99m-labeled for sentinel lymph node mapping of all solid tumors [29]. Also, in 2021, FDA has approved the usage of Technetium-99m-labeled Tilmanocept for pediatric sentinel lymph node mapping [30].

Although SLNB for HNSCC has been incorporated in the guidelines of National Comprehensive Cancer Network since 2014, Cramer et al. have noted that SLNB for HNSCC has not been widely accepted [31]. In 2018, Schilling et al. have published the guidelines for SLNB for HNSCC [32]. To further determine the role of SLNB in HNSCC, a multicenter randomized study using Technetium-99m-labeled Tilmanocept (Lymphoseek®) has been proposed based on several reasons. First, a randomized study may provide compelling level I evidence to guide treatment decisions [31]. Second, the role of SLNB in HNSCC should be evaluated to see if the procedure may reduce shoulder complications while preserving the rate of disease control [20]. Third, the randomized study will establish firmer ground to render management guidelines for SLNB in HNSCC [33]. Thus, a randomized Phase II–III trial of SLNB versus elective neck dissection for early-stage oral cavity cancer (NRG-HN006) under the auspice of NRG Oncology of the NCI National Clinical Trials Network has been developed with incorporation of quality of life characteristics for the study [34]. Since both melanoma and breast cancer have conducted randomized clinical trials to establish the validity of SLNB, it is appropriate that a well-planned randomized clinical trial for HNSCC to determine the utility of SLNB versus elective neck dissection should be conducted.

## Summary

In this review article, Technetium-99m-labeled Tilmanocept (Lymphoseek®) has described in detail of its molecular formula, its initial development as a radioisotope for detection of SLNs in melanoma and breast cancer and its successful application in melanoma, breast cancer and HNSCC clinical trials. Based on its safety and ability to detect SLNs satisfactorily, it has been approved by the FDA to use as a radioisotope for preoperative lymphoscintigraphy for identification of SLNs in melanoma, breast cancer and HNSCC.

## Abbreviations

SLN: Sentinel lymph node
SLNB: Sentinel lymph node biopsy
HNSCC: Head and neck squamous cell carcinoma
